# Disseminated Infection Caused by *Francisella philomiragia*, France, 2014

**DOI:** 10.3201/eid2112.150615

**Published:** 2015-12

**Authors:** Louis Kreitmann, Louis Terriou, David Launay, Yvan Caspar, René Courcol, Max Maurin, Nadine Lemaître

**Affiliations:** Centre Hospitalier Universitaire, Lille, France (L. Kreitmann, L. Terriou, D. Launay, R. Courcol, N. Lemaître);; Institut National de la Santé et de la Recherche Médicale (INSERM) U1019– Centre National de la Recherche Scientifique (CNRS) UMR8204,; Université de Lille-Nord de France, Lille (R. Courcol, N. Lemaître);; Centre Hospitalier Universitaire, Grenoble, France (Y. Caspar, M. Maurin);; CNRS/Université Joseph Fourier UMR5163, Université Grenoble Alpes, Grenoble (Y. Caspar, M. Maurin)

**Keywords:** Francisella philomiragia, Sweet syndrome, sepsis, pneumonia, respiratory infections, bacteria, zoonoses

**To the Editor:** *Francisella philomiragia* is a rare opportunistic pathogen. Only 17 cases of infection in humans have been reported over a 40-year period; 15 of these occurred in North America, mainly in persons who had near-drowning experiences and in patients with chronic granulomatous disease (*1*,*2*). We describe a case of *F. philomiragia* infection in a man in France who had the skin lesions of Sweet syndrome, characterized by nodules and pustules with dermal neutrophilic infiltration.

In 2014, a 58-year-old diabetic man with myeloproliferative disorders associated with Sweet syndrome (diagnosed in 2012), was hospitalized in the teaching hospital of Lille, France,with a 1-week history of fever (39°C) and cough. Physical examination showed inflammation around a central venous catheter, which was then removed. Blood, urine, and sputum samples that had been collected on admission were analyzed; leukocyte count was 5.83 × 10^9^ cells/L (neutrophils 79%) (reference range 4–10 × 10^9^ cells/L. Empirical treatment with piperacillin/tazobactam, vancomycin, and gentamicin was initiated. No pathogens were recovered from the samples, although the catheter was positive for *Staphylococcus epidermidis* and *Stenotrophomonas maltophilia*. These findings prompted us to replace piperacillin/tazobactam with ticarcillin/clavulanic acid in the treatment regimen. Despite a brief clinical improvement, the patient was highly febrile (40°C) 1 week after admission, and new blood samples were collected. A computed tomography scan of the thorax and abdomen revealed small nodules in the right lung and a single, large, hypodense lesion in the liver. Although histopathologic examination of a percutaneous liver biopsy specimen revealed multiple abscesses, no bacteria were observed after staining and culturing. Thus, the patient’s respiratory and hepatic symptoms were considered to be extracutaneous manifestations of Sweet syndrome.

Two aerobic cultures (BactAlert FAN medium; bioMérieux, Lyon, France) of blood samples drawn >48 hours after ticarcillin/clavulanic acid therapy was begun yielded oxidase-positive gram-negative rods after 4 days’ incubation at 37°C. These bacteria were identified as *F. philomiragia* in a matrix-assisted laser desorption/ionization time-of-flight mass spectrometry analysis (Bruker Daltonic, Bremen, Germany). Identification was confirmed by amplification and DNA sequencing of a portion of the 16S and 23S rRNA encoding genes as well as the intergenic region (*3*). On the basis of antimicrobial drug susceptibility testing results (performed 2 days after the positive culture), a combination of cefotaxime plus gentamicin was initiated (followed 3 days later by a regimen that included cefotaxime and ciprofloxacin). The patient’s symptoms resolved after 14 days of this treatment. Serum samples collected at admission and 2 weeks later were positive for IgM and IgG against *F. philomiragia* (titer 1:640 and 1:160, respectively, cutoff >1:20).

*F. philomiragia* is halophilic and appears to be ubiquitous in marine habitats in northern Europe (*4*). Thus, the patient may have been inoculated cutaneously with *F. philomiragia* through saltwater exposure because he lived near the coast of the North Sea and had bought and prepared locally caught fish and shellfish for his own consumption. The neutrophilic dermatosis lesions on the patient’s hands may have provided a portal of entry for *F. philomiragia*. Indeed, cutaneous inoculation of *F. philomiragia* has been reported in a patient after he was scratched by a crab (*2*). However, the patient we report could not recall ever having any local inflammation, skin ulcers, or subsequently enlarged lymph nodes after handling fish and shellfish. Although human cases of *F. philomiragia* infection are predominantly associated with saltwater, this organism also has been isolated from freshwater ponds, marshes, and warm springs in the United States (*5*,*6*). However, this patient did not recall contact with any of these aquatic environments.

In the few reported human cases of *F. philomiragia* infection, pneumonia was the most common clinical manifestation, as in the case we describe. Thus, inhalation of aerosols from a contaminated environment might constitute a noncutaneous route of *Francisella* spp. transmission. It is noteworthy that *Francisella*-like organisms were recovered from urban aerosol samples in Texas (*7*) and *F. guang*z*houensis* (which displays 95% nucleotide sequence identity with the 16S and 23S rRNA genes of *F. philomiragia*) was isolated from water in air-conditioning cooling towers (*8*). However, this patient did not recall ever having lived near sources of potential contaminated aerosols.

The patient could have been inoculated with *F. philomiragia* by arthropod bite (analogous to the tick bites that transmit the virulent species *F. tularensis*). To date, arthropod-based transmission *F. philomiragia* has not been suspected. However, *F. philomiragia* DNA was found in 19% of a sample of dog ticks (*Dermacentor reticulatus*) in France (*9*). This finding suggests that *D. reticulatus*, which is now broadly distributed across Europe because of global warming and increased travel with pets, may have a role in the life cycle and transmission of *F. philomiragia* (*10*). The patient did not own a dog and did not recall having had contact with dogs. However, his job (a municipal gardener) constituted a risk factor for tick bites in urban green spaces.

Although multiple points for *F. philomiragia* to enter this patient were suspected, none were laboratory confirmed. Further investigation is needed to better define the natural life cycle of this organism, especially the role of tick species in its transmission.
